# A phylogenetic generalized hidden Markov model for predicting alternatively spliced exons

**DOI:** 10.1186/1748-7188-1-14

**Published:** 2006-08-25

**Authors:** Jonathan E Allen, Steven L Salzberg

**Affiliations:** 1 Center for Bioinformatics and Computational Biology, University of Maryland Institute for Advanced Computer Studies, University of Maryland, College Park, MD 20742, USA; 2 Department of Computer Science, Johns Hopkins University, 3400 N. Charles Street, Baltimore, MD 21218, USA; 3 Department of Computer Science, University of Maryland, College Park, MD 20742, USA

## Abstract

**Background:**

An important challenge in eukaryotic gene prediction is accurate identification of alternatively spliced exons. Functional transcripts can go undetected in gene expression studies when alternative splicing only occurs under specific biological conditions. Non-expression based computational methods support identification of rarely expressed transcripts.

**Results:**

A non-expression based statistical method is presented to annotate alternatively spliced exons using a single genome sequence and evidence from cross-species sequence conservation. The computational method is implemented in the program ExAlt and an analysis of prediction accuracy is given for *Drosophila melanogaster*.

**Conclusion:**

ExAlt identifies the structure of most alternatively spliced exons in the test set and cross-species sequence conservation is shown to improve the precision of predictions. The software package is available to run on *Drosophila *genomes to search for new cases of alternative splicing.

## Background

High-throughput sequencing of expression data provides compelling evidence that the long held hypothesis "one gene produces one protein" is far less common than previously thought. Surveys from the human genome estimate that as many as 70% of human genes produce more than one transcribed form [1]. Examples are found in a variety of metazoan organisms confirming that a significant number of genes produce multiple distinct transcripts [2,3]. Alternative splicing is an important biological mechanism for producing multiple distinct transcripts from a single gene locus. Exon intron junctions are pieced together to produce differing mRNAs. In some cases alternative exon splicing leads to different functional proteins thereby increasing protein diversity. In other cases an alternatively spliced exon leads to non-functional mRNA, effectively regulating gene expression [3].

**Figure 1:**
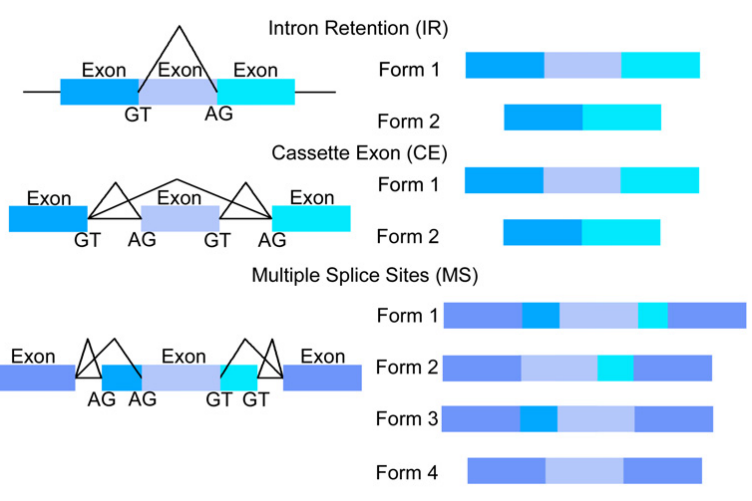
Three forms of alternative splicing: Intron Retention (IR), Cassette Exon (CE), and Multiple Splice sites (MS).

Given an input genomic sequence and the locations of gene regions, our goal is to find the functional exons originating from each gene locus, identifying their respective amino acid codons and splice sites. Figure 1 shows examples of alternatively spliced exons examined in this study: intron retention (IR), cassette exon (CE), and multiple splice sites (MS). Also considered are constitutive exons (CS), defined to be an exon included with the same splice site boundaries in all functional mRNA forms.

### Related work

Gene expression provides evidence for large numbers of alternatively spliced genes [4-10]. The most reliable high throughput evidence for alternative splicing comes from full length cDNAs, which are limited in coverage across all biological states. Expressed Sequence Tags (ESTs) supplement the coverage of full length cDNAs but still fail to capture all expressed forms [11,12]. Genomic sequence patterns can potentially be used to identify alternative splicing in less commonly expressed genes and recent work has focused on developing computational methods to predict alternative splicing without direct evidence of gene expression. This work is divided into two types: explicit and implicit alternative splicing prediction.

#### Explicit alternative splicing prediction

Sorek et al. looked at cassette exons in human and mouse and found a striking pattern of increased intron conservation distinct from constitutive exons [13]. A list of features were compiled including exon length, sequence conservation and k-mer counts [14,15], which were used in a support vector machine (SVM) [15] to classify cassette and constitutive exons. Yeo et al. developed a regularized least-squares classifier, called ACESCAN [16], to identify cassette exons in human/mouse orthologs using a similar feature set. A SVM cassette exon classifier was developed for *Caenorhabditis elegans *using only single species features and was extended to predict cassette exons in intron sequence [17]. *Drosophila melanogaster *exons matched to *Drosophila pseudoobscura *orthologs with conserved flanking intron sequence were observed by Philipps et al. to be enriched for alternatively spliced exons [18].

#### Implicit alternative splicing prediction

An alternative approach is to predict multiple overlapping gene structures, or a single gene structure overlapping existing alternative annotation. Explicit features of alternative splicing are not scored, but by virtue of having multiple overlapping high scoring gene structures, alternative splicing is implied. One method sampled paths [19] in the generalized hidden Markov Model (GHMM) of the single isoform gene finder SLAM [20]. Re-occurring overlapping high scoring parses were reported as candidates for alternative splicing. Another approach is to find an exon splicing pattern with the highest scoring alignment to profile hidden Markov models (profile-HMMs) [21]. The human genome was searched for cassette exons and intron retention events using a reference annotation [22]. Predicted gene structures with scores exceeding the reference gene structure were inferred to be examples of alternative splicing.

The work most similar to the model introduced in this article is the pair-HMM UNCOVER [23], which finds exons in sequence annotated as introns and was tested on human/mouse intron pairs. Unlike the cassette exon classification methods [15-17], models were trained using examples of protein coding exons without explicitly distinguishing between constitutive exons and cassette exons. Since the input sequence is assumed to be an intron, predicted exons are inferred to be alternatively spliced.

The method presented in this article extends the GHMMs used in single isoform gene finding [24] to explicitly model features of alternative and constitutive exons. The features of the explicit alternative splicing prediction methods: k-mer counts, exon lengths, and sequence conservation are used to predict multiple splice sites and intron retention events along with cassette exons and constitutive exons. Cross-species sequence conservation is incorporated using components of the single isoform phylogenetic HMM gene finders [25-27]. The phylogenetic shadowing principle is used to assume a multiple sequence alignment can be obtained from closely related species [28]. In contrast, the pair-HMM method simultaneously predicts a pairwise alignment and the exon structure making it potentially better suited to incorporate a difficult to align, more distantly related organism. Conservation from greater evolutionary distances may improve discriminative power in identify functional nucleotides, but with the potential trade off of detecting a smaller set of conserved alternative splicing events [29].

The remainder of the article describes our computational prediction model and reports on prediction accuracy in *Drosophila melanogaster*.

## Results and discussion

### Graphical model of alternative splicing

The prediction model is designed to predict multiple overlapping exons in a sequence believed to contain a single exon or intron, rather than the complete gene from start codon to stop codon. Input is expected to be a target sequence previously annotated by a single-isoform gene annotation tool such as a gene finder, cDNA alignment or some other annotation source. In cases where the input sequence contains untranslated regions, it is assumed that the coding boundary is known. Thus, the problem of translation start/stop site prediction is not addressed here.

**Figure 2:**
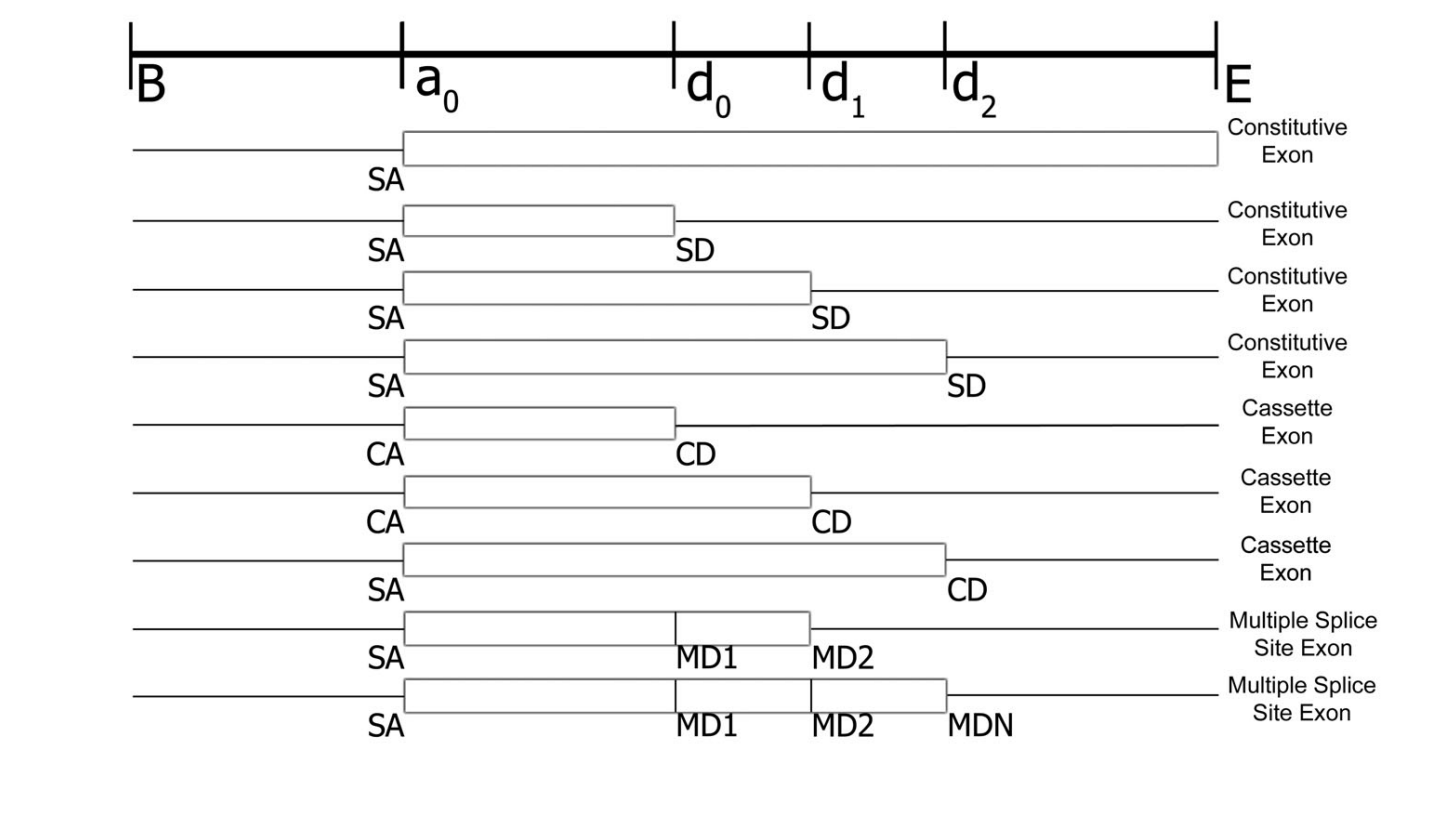
Alternative and single isoform exon candidates. Four splice sites are shown, one acceptor *a*_0_, and three donor sites, *d*_0_, *d*_1_, and *d*_2_, and the begin (B) and end (E) of the input sequence. There are four candidate constitutive exons (Constitutive Exon), three candidate cassette exons (Cassette Exon), and candidate exons with multiple functional donor sites (Multiple Splice Site Exon). See text for a description of the splice types: single acceptor (SA), cassette acceptor (CA), single donor (SD), cassette donor (CD), multiple donor 1 (MD1), multiple donor 2 (MD2), and multiple donor above two in number (MDN).

Alternative splicing increases the number of candidate acceptor/donor pairs compared to the constitutive exon equivalent. Figure 2 shows four candidate splice sites, an acceptor site *a*_0_, and three donor sites *d*_0_, *d*_1_, and *d*_2_. In a single isoform gene finder, only one of the four exons labeled constitutive in Figure 2 represent a viable exon. Allowing for alternative splicing means all three donors sites are potentially functional. For example, in Figure 2, the eighth candidate splicing type from the top has two functional donor sites *d*_0_(marked *MD*1) and *d*_1 _(marked *MD*2), leading to two different functional exons. More than two functional donor or acceptor sites can occur leading to a model of unbounded size. The combinatorial possibilities are reduced to a finite number using one symbol for each splice type to represent functional splice sites over 2 in number. For example, MDN is the symbol used to represent the third functional donor site, *d*_2 _in Figure 2.

Donor sites are divided into five types: single functional constitutive donor *SD*, alternative donor for cassette exons *CD *and multiple functional donors *MD*1, *MD*2, and *MDN*. *MD*1 is the left most functional donor, *MD2 *is the donor immediately downstream of *MD1*, and *MDN *represents additional downstream donors. The classification scheme similarly extends to acceptor sites: *SA *(single constitutive acceptor), *CA *(acceptor for cassette exon), *MA1 *(first multiple acceptor), *MA2 *(second multiple acceptor), and *MAN *(multiple acceptors greater than 2).

The intron retention splice site labeled GT in Figure 1 (the 5' end of the retained intron) forms the basis of the splicing types: *SD-IR*, *MD1-IR*, *MD2-IR*, and *MDN-IR*. The intron retention acceptor labeled AG in Figure 1 (3' end of the retained intron) forms the basis for the splicing types: *SA-IR*, *MA1-IR*, *MA2-IR*, and *MAN-IR*. There are five end of sequence conditions: beginning of the sequence (Beg), end of a constitutive intron (END-INTRON_*C*_), end of an alternative intron (END-INTRON_*A*_), end of a constitutive exon (END-EXON_*C*_), and end of an alternative exon (END-EXON_*A*_).

**Figure 3:**
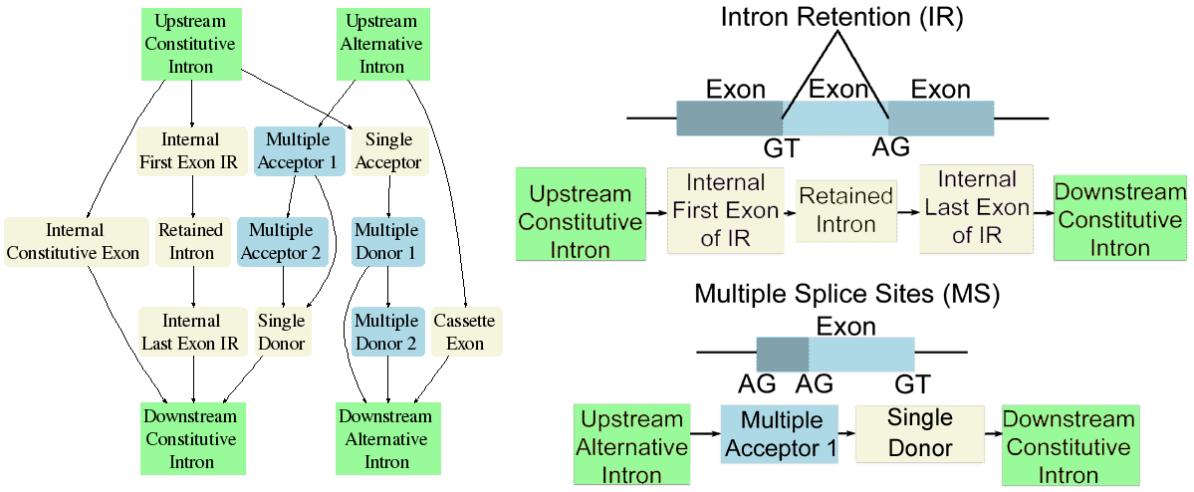
Left image shows a portion of the graphical model for alternatively spliced exons. The right side of the figure shows two examples of parsing a target sequence. The top right example parses an intron retention sequence and the bottom right example parses a multiple splice site sequence. Blue states output partial subsequence of alternatively spliced exons, beige states are exons beginning with an acceptor and ending with a donor. Green states are introns.

Splice sites and end of sequence conditions are called signals and ordered signal pairs define the exon/intron intervals in an alternative exon splicing model. Figure 3 shows a portion of the model and two example sets of states aligned to genomic sequence. The model in Figure 3 predicts alternative splicing in internal exons for the three splicing types in Figure 1 plus constitutive exons. The states represent sequence intervals between pairs of signals. The top right example in Figure 3 shows an initial "Upstream Constitutive Intron" state between signal pair (Beg, SD), which marks an intron proximal to a constitutive splice site followed by states for each downstream exon interval: "Internal First Exon of IR" (SD,SD-IR), "Retained Intron" (SD-IR,SA-IR), and "Internal Last Exon of IR" (SA-IR,SD), ending in the "Downstream Constitutive Intron" state (SD,END-INTRON*c*). The bottom right example in Figure 3 shows "Upstream Alternative Intron" (Beg,MA1), "Multiple Acceptor 1" (MA1,MA2), "Single Donor" (MA2,SD), and "Downstream Constitutive Intron" (SD, END-INTRON_*C*_). States not shown in Figure 3 model rarer forms of splicing, including combinations of alternative splicing events and splicing in exons at the end of genes. The complete model is given in the Methods section.

#### Phylogenetic generalized hidden Markov model definition

A phylogenetic generalized hidden Markov model (PGHMM) extends a model described in the single isoform gene finder Shadower [27]. The method described here models higher order nucleotide dependencies and is applied to the alternative exon splicing model introduced in Figure 3. An input multiple sequence alignment *X *= *S*_0_,..., *S*_*m *_includes the target sequence *S*_0 _and *m *informant species. X[k] is the kth column in X and X[i,j] are the columns from position i to j inclusive, the PGHMM is defined to be a 7 tuple, (*Q*, *π*, Σ, *R*, ψ, *O*, *L*):

• *Q *– the set of states with states *q*, *q*'. ∈ *Q*

• *P*_*π*_(*q*) – the probability of beginning in state q

• Σ – the set of nucleotides {*A*, *C*, *G*, *T*} emitted in the model

• *P*_*R*_(*q*|*q*') – the transition probabilities from state *q*' to state *q*

• *ψ *– the set of phylogenetic parameters

• *P*_*O *_(*X *[*i*, *j*]|*q*, *ψ*) – the probability of emitting sequence alignment columns from *i *to *j *in state *q *using phylogenetic parameter set *ψ*

• *P*_*L*, *q *_(*j *- *i *+ 1) – the probability of the state *q *emitting the series of columns of length *j *- *i *+ 1

The parse of multiple sequence alignment X is a series of partitions *t *= (*t*_0_, *t*_1_, ..., *t*_*n*_), with state *q*_*i *_outputting a contiguous series of columns in X from position *b*_*i *_to *e*_*i *_inclusive in partition *t*_*i *_= (*b*_*i*_, *e*_*i*_, *q*_*i*_). The parse spans the entire multiple sequence alignment *X *so that *b*_*i *+ 1 _= *e*_*i *_+ 1. The joint probability between parse *t *and sequence *X *is:

P(t,X)=PO(X[b0,e0]|q0,ψ)×Pπ(q0)×PL,q0(e0+1)×∏i=1nPO(X[bi,ei]|qi,ψ)×PR(qi|qi−1)×PL,qi(ei−bi+1).
MathType@MTEF@5@5@+=feaafiart1ev1aaatCvAUfKttLearuWrP9MDH5MBPbIqV92AaeXatLxBI9gBaebbnrfifHhDYfgasaacH8akY=wiFfYdH8Gipec8Eeeu0xXdbba9frFj0=OqFfea0dXdd9vqai=hGuQ8kuc9pgc9s8qqaq=dirpe0xb9q8qiLsFr0=vr0=vr0dc8meaabaqaciaacaGaaeqabaqabeGadaaakeaafaqabeWabaaabaGaemiuaaLaeiikaGIaemiDaqNaeiilaWIaemiwaGLaeiykaKIaeyypa0dabaGaemiuaa1aaSbaaSqaaiabd+eapbqabaGccqGGOaakcqWGybawcqGGBbWwcqWGIbGydaWgaaWcbaGaeGimaadabeaakiabcYcaSiabdwgaLnaaBaaaleaacqaIWaamaeqaaOGaeiyxa0LaeiiFaWNaemyCae3aaSbaaSqaaiabicdaWaqabaGccqGGSaaliiGacqWFipqEcqGGPaqkcqGHxdaTcqWGqbaudaWgaaWcbaGae8hWdahabeaakiabcIcaOiabdghaXnaaBaaaleaacqaIWaamaeqaaOGaeiykaKIaey41aqRaemiuaa1aaSbaaSqaaiabdYeamjabcYcaSiabdghaXnaaBaaameaacqaIWaamaeqaaaWcbeaakiabcIcaOiabdwgaLnaaBaaaleaacqaIWaamaeqaaOGaey4kaSIaeGymaeJaeiykaKIaey41aqlabaWaaebmaeaacqWGqbaudaWgaaWcbaGaem4ta8eabeaaaeaacqWGPbqAcqGH9aqpcqaIXaqmaeaacqWGUbGBa0Gaey4dIunakiabcIcaOiabdIfayjabcUfaBjabdkgaInaaBaaaleaacqWGPbqAaeqaaOGaeiilaWIaemyzau2aaSbaaSqaaiabdMgaPbqabaGccqGGDbqxcqGG8baFcqWGXbqCdaWgaaWcbaGaemyAaKgabeaakiabcYcaSiab=H8a5jabcMcaPiabgEna0kabdcfaqnaaBaaaleaacqWGsbGuaeqaaOGaeiikaGIaemyCae3aaSbaaSqaaiabdMgaPbqabaGccqGG8baFcqWGXbqCdaWgaaWcbaGaemyAaKMaeyOeI0IaeGymaedabeaakiabcMcaPiabgEna0kabdcfaqnaaBaaaleaacqWGmbatcqGGSaalcqWGXbqCdaWgaaadbaGaemyAaKgabeaaaSqabaGccqGGOaakcqWGLbqzdaWgaaWcbaGaemyAaKgabeaakiabgkHiTiabdkgaInaaBaaaleaacqWGPbqAaeqaaOGaey4kaSIaeGymaeJaeiykaKIaeiOla4caaaaa@A1B6@

Each interval from *b *to *e *begins and ends with a signal covering a fixed length window, *Sig*_*b *_and *Sig*_*e *_respectively. The donor signal window width *W*_*don*_, is set to 9 for all donor types (SD, CD, MD1, MD2, MDN, SD-IR, MD1-IR, MD2-IR, MDN-IR). When the window covers columns *X *[*k*, *k *+ 8] from *k *to *k *+ 8 the consensus splice site is in subsequence *S*_0 _[*k *+ 3, *k *+ 4] = *GT*. The acceptor window width *W*_*acc*_, is set to 24 for all acceptor types (SA, CA, MA1, MA2, MAN, SA-IR, MA1-IR, MA2-IR, MAN-IR), covering columns *X*[*k *- 23, *k*] from k-23 to k with consensus splice site in subsequence *S*_0_[*k *- 3, *k *- 2] = *AG*. The beginning and ending sequence signals set the window parameter *W*_*Sig *_to 0 since non splice site signals are not explicitly modeled.

State *q *emitting columns *X*[*b*, *e*] from *b *to *e *models the downstream signal *Sig*_*e *_but excludes the upstream signal *Sig*_*b*_. For example, when *q *= *Multiple Donor *1, q outputs columns between two donor sites, *Sig*_*b *_= *MD*1 and *Sig*_*e *_= *MD*2. The exon interval is scored from *b *to e-*W*_*don *_- 1 inclusive and the donor columns are scored from *e *- *W*_*don *_to *e *inclusive. The upstream donor site window *MD*1 spans the interval *b *- *W*_*don *_- 1 to *b *- 1 and is scored in the previous state.

The probability of a state emitting a series of columns becomes:

PO(X[b,e]|q,ψ)=∏k=beP(X[k]|SelectModel(X,e,k,z,q,ψ))
 MathType@MTEF@5@5@+=feaafiart1ev1aaatCvAUfKttLearuWrP9MDH5MBPbIqV92AaeXatLxBI9gBaebbnrfifHhDYfgasaacH8akY=wiFfYdH8Gipec8Eeeu0xXdbba9frFj0=OqFfea0dXdd9vqai=hGuQ8kuc9pgc9s8qqaq=dirpe0xb9q8qiLsFr0=vr0=vr0dc8meaabaqaciaacaGaaeqabaqabeGadaaakeaacqWGqbaudaWgaaWcbaGaem4ta8eabeaakiabcIcaOiabdIfayjabcUfaBjabdkgaIjabcYcaSiabdwgaLjabc2faDjabcYha8jabdghaXjabcYcaSGGaciab=H8a5jabcMcaPiabg2da9maarahabaGaemiuaaLaeiikaGIaemiwaGLaei4waSLaem4AaSMaeiyxa0LaeiiFaWNaem4uamLaemyzauMaemiBaWMaemyzauMaem4yamMaemiDaqNaemyta0Kaem4Ba8MaemizaqMaemyzauMaemiBaWMaeiikaGIaemiwaGLaeiilaWIaemyzauMaeiilaWIaem4AaSMaeiilaWIaemOEaONaeiilaWIaemyCaeNaeiilaWIae8hYdKNaeiykaKIaeiykaKcaleaacqWGRbWAcqGH9aqpcqWGIbGyaeaacqWGLbqza0Gaey4dIunaaaa@6C9F@

The probability of emitting each column in the alignment is defined by a sequence model returned by *SelectModel*(*X*, *e*, *k*, *z*, *q*, *ψ*). The current position *k *in alignment X, the end position of the scored interval (*e*), current state q, protein coding phase *z*, and phylogenetic parameters *ψ *determine the choice of sequence models. If *q *is an exon state and k is within the coding region, the coding phase *z *is 0, 1 or 2 and -1 otherwise. When q is an exon state and k is outside the coding region, an untranslated exon region is implied. The sequence models are divided into three "template" categories, MSige
 MathType@MTEF@5@5@+=feaafiart1ev1aaatCvAUfKttLearuWrP9MDH5MBPbIqV92AaeXatLxBI9gBaebbnrfifHhDYfgasaacH8akY=wiFfYdH8Gipec8Eeeu0xXdbba9frFj0=OqFfea0dXdd9vqai=hGuQ8kuc9pgc9s8qqaq=dirpe0xb9q8qiLsFr0=vr0=vr0dc8meaabaqaciaacaGaaeqabaqabeGadaaakeaacqWGnbqtdaWgaaWcbaGaem4uamLaemyAaKMaem4zaC2aaSbaaWqaaiabdwgaLbqabaaaleqaaaaa@3367@, *M*_*codon*_, and *M*_*non-coding *_and an instance of one of these three types is returned by the function:

*SelectModel *(*X*, *e*, *k*, *z*, *q*, *ψ*) =

{MSige(X,k,e,ψ)k>e−WSigeMcodon(X,k,z,ψ)z≠−1Mnon−coding(X,k,ψ)otherwise}
 MathType@MTEF@5@5@+=feaafiart1ev1aaatCvAUfKttLearuWrP9MDH5MBPbIqV92AaeXatLxBI9gBaebbnrfifHhDYfgasaacH8akY=wiFfYdH8Gipec8Eeeu0xXdbba9frFj0=OqFfea0dXdd9vqai=hGuQ8kuc9pgc9s8qqaq=dirpe0xb9q8qiLsFr0=vr0=vr0dc8meaabaqaciaacaGaaeqabaqabeGadaaakeaadaGadeqaauaabeqadiaaaeaacqWGnbqtdaWgaaWcbaGaem4uamLaemyAaKMaem4zaC2aaSbaaWqaaiabdwgaLbqabaaaleqaaOGaeiikaGIaemiwaGLaeiilaWIaem4AaSMaeiilaWIaemyzauMaeiilaWccciGae8hYdKNaeiykaKcabaGaem4AaSMaeyOpa4JaemyzauMaeyOeI0Iaem4vaC1aaSbaaSqaaiabdofatjabdMgaPjabdEgaNnaaBaaameaacqWGLbqzaeqaaaWcbeaaaOqaaiabd2eannaaBaaaleaacqWGJbWycqWGVbWBcqWGKbazcqWGVbWBcqWGUbGBaeqaaOGaeiikaGIaemiwaGLaeiilaWIaem4AaSMaeiilaWIaemOEaONaeiilaWIae8hYdKNaeiykaKcabaGaemOEaONaeyiyIKRaeyOeI0IaeGymaedabaGaemyta00aaSbaaSqaaiabd6gaUjabd+gaVjabd6gaUjabgkHiTiabdogaJjabd+gaVjabdsgaKjabdMgaPjabd6gaUjabdEgaNbqabaGccqGGOaakcqWGybawcqGGSaalcqWGRbWAcqGGSaalcqWFipqEcqGGPaqkaeaacqWGVbWBcqWG0baDcqWGObaAcqWGLbqzcqWGYbGCcqWG3bWDcqWGPbqAcqWGZbWCcqWGLbqzaaaacaGL7bGaayzFaaaaaa@85B2@

In theory, each state could maintain separate sequence models. For example, the "Internal First Exon of IR" state could model codon usage separately from the "Internal Last Exon of IR" state. In practice, this results in far too many parameters to estimate given training data sizes. Instead the states are tied to 10 candidate sequence models returned by *SelectModel*. The models are listed with the analogous Markov models commonly used in single isoform *ab initio *gene finders.

• *M*_*non-coding *_(*X*, *k*, *ψ*). 3rd order homogeneous Markov model: *P*(*S*_0_[*k*]|*S*_0_[*k *- 3, *k *- 1])

          - *M*_*AUTR*_(*X*, *k*, *ψ*) – alternative 5'/3' untranslated region (AUTR)

          - *M*_*CUTR *_(*X*, *k*, *ψ*) – constitutive 5'/3' untranslated region (CUTR)

          - *M*_*AI *_(*X*, *k*, *ψ*) – alternative intron (AI)

          - *M*_*CI *_(*X*, *k*, *ψ*) – constitutive intron (CI)

• *M*_*codon *_(*X*, *k*, *z*, *ψ*). 3rd order inhomogeneous 3-periodic Markov model: *P*^*z *^(*S*_0_[*k*]|*S*_0_[*k *- 3, *k *- 1])

          - *M*_*AE *_(*X*, *k*, *z*, *ψ*) – alternative exon (AE)

          - *M*_*CE *_(*X*, *k*, *z*, *ψ*) – constitutive exon (CE)

• *M*_*don *_(*X*, *k*, *e*, *ψ*). 1st order inhomogeneous Markov model (WAM): *P*^9-(*e *- *k*) ^(*S*_0_[*k*]|*S*_0_[*k *- 1])

          - *M*_*SD *_(*X*, *k*, *e*, *ψ*) – constitutive/single donor (SD)

          - *M*_*AD *_(*X*, *k*, *e*, *ψ*) – alternative donor (AD) (covers all alternative donor types)

• *M*_*acc *_(*X*, *k*, *e*, *ψ*). 1st order inhomogeneous Markov model (WAM): *P*^24-(*e *- *k*) ^(*S*[*k*]|*S*[*k *- 1])

          - *M*_*SA *_(*X*, *k*, *e*, *ψ*)- constitutive/single acceptor (SA)

          - *M*_*AA *_(*X*, *k*, *e*, *ψ*)- alternative acceptor (AA) (covers all alternative acceptor types)

### From Markov models to evolutionary models

The key difference between our implementation and a single isoform *ab initio *gene finder is two fold: 1) separate models are maintained for the two splicing types: alternative and constitutive, and 2) the nucleotide dependencies are modeled using an evolutionary framework. The choice to separate sequence models for the two splicing types is motivated by the previous work explicitly classifying alternatively spliced exons and by the hypothesis that a splice site can be activated or deactivated with proximal splicing factors binding to the pre-mRNA sequence to interact directly (or indirectly) with the Spliceosome. The presence of splicing factors in conjunction with the characteristics of the splice site is expected to determine splice site usage. There is growing evidence that many alternative splicing events follow this model [30,31]. 

**Figure 4:**
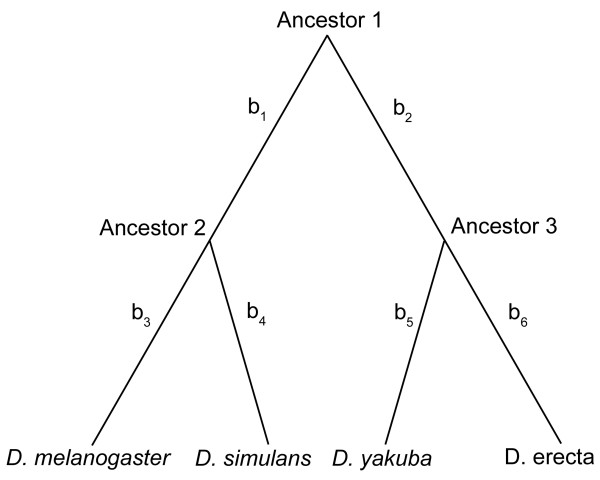
Phylogenetic tree for four species of *Drosophila*. Each branch *i *has a branch length of *b*_*i*_.

Each sequence model estimates the probability of emitting column *X*[*k*] using a phylogenetic tree. Figure 4 shows a schematic of the phylogenetic tree for four *Drosophila *species used in testing. The goal is to compute the probability of the observed column having evolved from a common ancestral sequence. For the tree in Figure 4, assume the ancestral base at the root ("Ancestor 1") to be A and the descendant node ("Ancestor 2") to be C. The probability of A evolving to C is computed using a nucleotide substitution model. The HYK model [32] was chosen for the use of three features: distinguished transition/transversion mutation events (assumed to be a fixed parameter), a nucleotide equilibrium model, and the evolutionary time interval defined by the tree branch length. The probability of emitting column *X*[*k*] is found by computing, in linear time with respect to the number of nodes in the tree, the probability of all possible ancestral sequences having evolved into the observed column using the Felsenstein phylogenetic tree scoring procedure [33].

The nucleotide equilibrium parameters of the HYK model are naturally suited to incorporate the nucleotide bias found in the different sequence models (e.g. donor, codon, etc.). With a multiple sequence alignment as input, an intuitive extension to the *ab initio *Markov model is to use the preceding *o *bases from each input sequence to estimate the likelihood of the current nucleotide (where *o *is the order of the Markov model). For example, in Figure 4, estimating the probability of nucleotide C at "Ancestor 2" having evolved from nucleotide A at "Ancestor 1", should reflect the nucleotide equilibrium of *D. melanogaster *and *D. simulans*, given the *o *previous bases in the input alignment for the two species. Similarly, estimating the probability of the root ancestral base being A (Ancestor 1) should reflect the nucleotide equilibrium among all four species given the *o *previous nucleotides in the input alignment from all four species. If *d*_*v *_is the number of descendants at node *v*, the number of parameters is ∑v4dv+o+1
 MathType@MTEF@5@5@+=feaafiart1ev1aaatCvAUfKttLearuWrP9MDH5MBPbIqV92AaeXatLxBI9gBaebbnrfifHhDYfgasaacH8akY=wiFfYdH8Gipec8Eeeu0xXdbba9frFj0=OqFfea0dXdd9vqai=hGuQ8kuc9pgc9s8qqaq=dirpe0xb9q8qiLsFr0=vr0=vr0dc8meaabaqaciaacaGaaeqabaqabeGadaaakeaadaaeqaqaaiabisda0maaCaaaleqabaGaemizaq2aaSbaaWqaaiabdAha2bqabaWccqGHRaWkcqWGVbWBcqGHRaWkcqaIXaqmaaaabaGaemODayhabeqdcqGHris5aaaa@3835@ (*v *enumerating over all nodes in the tree) leaving too many parameters to reliably estimate, given the current limits on training sizes.

Frequency counts obtained from each organism independently, reduce the parameter size for each sequence model to (*m *+ 1) × 4^*o *+ 1 ^(where *m *+ 1 is the number of organisms). Let S′0,…,S′m′
 MathType@MTEF@5@5@+=feaafiart1ev1aaatCvAUfKttLearuWrP9MDH5MBPbIqV92AaeXatLxBI9gBaebbnrfifHhDYfgasaacH8akY=wiFfYdH8Gipec8Eeeu0xXdbba9frFj0=OqFfea0dXdd9vqai=hGuQ8kuc9pgc9s8qqaq=dirpe0xb9q8qiLsFr0=vr0=vr0dc8meaabaqaciaacaGaaeqabaqabeGadaaakeaacuWGtbWugaqbamaaBaaaleaacqaIWaamaeqaaOGaeiilaWIaeSOjGSKaeiilaWIafm4uamLbauaadaWgaaWcbaGafmyBa0Mbauaaaeqaaaaa@34C3@ be the sequences descendant from node *v*. If *c*(S′i
 MathType@MTEF@5@5@+=feaafiart1ev1aaatCvAUfKttLearuWrP9MDH5MBPbIqV92AaeXatLxBI9gBaebbnrfifHhDYfgasaacH8akY=wiFfYdH8Gipec8Eeeu0xXdbba9frFj0=OqFfea0dXdd9vqai=hGuQ8kuc9pgc9s8qqaq=dirpe0xb9q8qiLsFr0=vr0=vr0dc8meaabaqaciaacaGaaeqabaqabeGadaaakeaacuWGtbWugaqbamaaBaaaleaacqWGPbqAaeqaaaaa@2F6E@[*k *- *o*, *k *- 1], *n*) returns the number of times each nucleotide *n *∈ {*A*, *C*, *G*, *T*} was observed to follow the substring S′i
 MathType@MTEF@5@5@+=feaafiart1ev1aaatCvAUfKttLearuWrP9MDH5MBPbIqV92AaeXatLxBI9gBaebbnrfifHhDYfgasaacH8akY=wiFfYdH8Gipec8Eeeu0xXdbba9frFj0=OqFfea0dXdd9vqai=hGuQ8kuc9pgc9s8qqaq=dirpe0xb9q8qiLsFr0=vr0=vr0dc8meaabaqaciaacaGaaeqabaqabeGadaaakeaacuWGtbWugaqbamaaBaaaleaacqWGPbqAaeqaaaaa@2F6E@[*k *- *o*, *k *- 1] at position k in a training alignment, the nucleotide equilibrium at node *v *for each nucleotide *n *is:

∑i=0m′c(S′i[k−o,k−1],n)/(∑i=0m′∑n′∈{A,C,G,T}c(S′i[k−o,k−1],n′))
 MathType@MTEF@5@5@+=feaafiart1ev1aaatCvAUfKttLearuWrP9MDH5MBPbIqV92AaeXatLxBI9gBaebbnrfifHhDYfgasaacH8akY=wiFfYdH8Gipec8Eeeu0xXdbba9frFj0=OqFfea0dXdd9vqai=hGuQ8kuc9pgc9s8qqaq=dirpe0xb9q8qiLsFr0=vr0=vr0dc8meaabaqaciaacaGaaeqabaqabeGadaaakeaadaaeWbqaaiabdogaJjabcIcaOiqbdofatzaafaWaaSbaaSqaaiabdMgaPbqabaaabaGaemyAaKMaeyypa0JaeGimaadabaGafmyBa0Mbauaaa0GaeyyeIuoakiabcUfaBjabdUgaRjabgkHiTiabd+gaVjabcYcaSiabdUgaRjabgkHiTiabigdaXiabc2faDjabcYcaSiabd6gaUjabcMcaPiabc+caViabcIcaOmaaqahabaWaaabuaeaacqWGJbWycqGGOaakcuWGtbWugaqbamaaBaaaleaacqWGPbqAaeqaaOGaei4waSLaem4AaSMaeyOeI0Iaem4Ba8MaeiilaWIaem4AaSMaeyOeI0IaeGymaeJaeiyxa0LaeiilaWIafmOBa4MbauaacqGGPaqkcqGGPaqkaSqaaiqbd6gaUzaafaGaeyicI4Saei4EaSNaemyqaeKaeiilaWIaem4qamKaeiilaWIaem4raCKaeiilaWIaemivaqLaeiyFa0habeqdcqGHris5aaWcbaGaemyAaKMaeyypa0JaeGimaadabaGafmyBa0Mbauaaa0GaeyyeIuoaaaa@7121@

Tree branch lengths are assumed to be fixed, but functional sequence elements are expected to exhibit a slower rate of substitution. Each sequence model maintains substitution rates to either expand the branch lengths of the tree (for rates greater than 1) or contract the branch lengths. Longer branch lengths have the effect of allowing mutations to accumulate in a column without incurring a scoring penalty, whereas, shorter branch lengths reward perfectly preserved columns. The codon, intron, and untranslated region states use two substitution rates, one rate for when the input column is conserved (no mutations observed) and a second rate when the column is not conserved. If a mutation is observed in a codon where the encoded amino acid is preserved, the higher substitution rate is selected to better accept the mutation. In the case of splice sites, each base is assumed to be subject to selective pressure and a single rate is used. An optimal parse of multiple sequence alignment *X *is found taking the log ratio of a state emitting the columns in X versus an equivalent model assuming all nucleotides are equally probable (the "background" state). Using a dynamic programming matrix *D*(*j*, *q*) initialized to

log(PO(X[0,j]|q,ψ)×Pπ(q)×PL,q(j+1)PO(X[0,j]|background,ψ))
 MathType@MTEF@5@5@+=feaafiart1ev1aaatCvAUfKttLearuWrP9MDH5MBPbIqV92AaeXatLxBI9gBaebbnrfifHhDYfgasaacH8akY=wiFfYdH8Gipec8Eeeu0xXdbba9frFj0=OqFfea0dXdd9vqai=hGuQ8kuc9pgc9s8qqaq=dirpe0xb9q8qiLsFr0=vr0=vr0dc8meaabaqaciaacaGaaeqabaqabeGadaaakeaaieGacqWFSbaBcqWFVbWBcqWFNbWzcqGGOaakdaWcaaqaaiabdcfaqnaaBaaaleaacqWGpbWtaeqaaOGaeiikaGIaemiwaGLaei4waSLaeGimaaJaeiilaWIaemOAaOMaeiyxa0LaeiiFaWNaemyCaeNaeiilaWccciGae4hYdKNaeiykaKIaey41aqRaemiuaa1aaSbaaSqaaiab+b8aWbqabaGccqGGOaakcqWGXbqCcqGGPaqkcqGHxdaTcqWGqbaudaWgaaWcbaGaemitaWKaeiilaWIaemyCaehabeaakiabcIcaOiabdQgaQjabgUcaRiabigdaXiabcMcaPaqaaiabdcfaqnaaBaaaleaacqWGpbWtaeqaaOGaeiikaGIaemiwaGLaei4waSLaeGimaaJaeiilaWIaemOAaOMaeiyxa0LaeiiFaWNaemOyaiMaemyyaeMaem4yamMaem4AaSMaem4zaCMaemOCaiNaem4Ba8MaemyDauNaemOBa4MaemizaqMaeiilaWIae4hYdKNaeiykaKcaaiabcMcaPaaa@7432@

entries for each nucleotide *j *and state *q *are assigned a value:

D(j,q)=maxi,q′log(PO(X[i,j]|q,ψ)×PT(q|q′)×PL,q(j−i+1)PO(X[i,j]|background,ψ)+D(i,q′)
 MathType@MTEF@5@5@+=feaafiart1ev1aaatCvAUfKttLearuWrP9MDH5MBPbIqV92AaeXatLxBI9gBaebbnrfifHhDYfgasaacH8akY=wiFfYdH8Gipec8Eeeu0xXdbba9frFj0=OqFfea0dXdd9vqai=hGuQ8kuc9pgc9s8qqaq=dirpe0xb9q8qiLsFr0=vr0=vr0dc8meaabaqaciaacaGaaeqabaqabeGadaaakeaafaqabeGabaaabaGaemiraqKaeiikaGIaemOAaOMaeiilaWIaemyCaeNaeiykaKIaeyypa0dabaacbiGae8xBa0Mae8xyaeMae8hEaG3aaSbaaSqaaiabdMgaPjabcYcaSiqbdghaXzaafaaabeaakiab=XgaSjab=9gaVjab=DgaNjabcIcaOmaalaaabaGaemiuaa1aaSbaaSqaaiabd+eapbqabaGccqGGOaakcqWGybawcqGGBbWwcqWGPbqAcqGGSaalcqWGQbGAcqGGDbqxcqGG8baFcqWGXbqCcqGGSaaliiGacqGFipqEcqGGPaqkcqGHxdaTcqWGqbaudaWgaaWcbaGaemivaqfabeaakiabcIcaOiabdghaXjabcYha8jqbdghaXzaafaGaeiykaKIaey41aqRaemiuaa1aaSbaaSqaaiabdYeamjabcYcaSiabdghaXbqabaGccqGGOaakcqWGQbGAcqGHsislcqWGPbqAcqGHRaWkcqaIXaqmcqGGPaqkaeaacqWGqbaudaWgaaWcbaGaem4ta8eabeaakiabcIcaOiabdIfayjabcUfaBjabdMgaPjabcYcaSiabdQgaQjabc2faDjabcYha8jabdkgaIjabdggaHjabdogaJjabdUgaRjabdEgaNjabdkhaYjabd+gaVjabdwha1jabd6gaUjabdsgaKjabcYcaSiab+H8a5jabcMcaPaaacqGHRaWkcqWGebarcqGGOaakcqWGPbqAcqGGSaalcuWGXbqCgaqbaiabcMcaPaaaaaa@8FC3@

Exons are recovered from the parse ending at the highest scoring entry max_*q *_*D*(*N *- 1, *q*) where *N *is the length of X. The runtime of the algorithm is *O*(|*Q*|^2 ^× *N*^2^) where |*Q*| is the number of states in the model. The PGHMM can easily be transformed to a single species alternative exon predictor by assuming a single input sequence and single node phylogenetic tree. The evolutionary models reduce to the single sequence Markov model equivalents and are used to measure the impact of sequence conservation on prediction performance.

### Experiments

The alternative exon splicing model was implemented in a program called ExAlt and tested on a target genome – *Drosophila melanogaster *using three informant species: *Drosophila simulans, Drosophila yakuba*, and *Drosophila erecta*. This study focuses on the three most closely related species to *D. melanogaster *(with available genomic data) to avoid using inaccurate multiple sequence alignments, which can occur when dealing with more distantly related species. Testing is based on 1339 *D. melanogaster *exons from 1160 gene loci. 572 of the original 600 alternatively spliced test exons (95%) were aligned to at least one of the three informant species and 767 of 777 constitutive exons (99%) were aligned to at least one of the three informant species. As an option, ExAlt predicts exons in the absence of alignment evidence; however, the candidate exons with no cross-species sequence conservation left too small a data set (3% = 38/1377) to make meaningful comparisons between performance on exons with and without detectable cross-species conservation. Therefore, the remaining 97% of the exons showing some cross-species sequence conservation were selected to evaluate the impact of sequence conservation on prediction performance, with the understanding that additional work will be needed (as more data becomes available) to analyze prediction performance in the non-conserved exons.

The goal of the experiments was to test ExAlt's ability to take a single input sequence presumed to contain an exon and correctly predict all of the exon/intron boundaries. The experiments were constructed to measure the impact of using gene structure information and cross species sequence conservation on prediction performance. ExAlt outputs exon coordinates and exon splicing type labels. 60% of the data (selected at random) was used to evaluate sequence conservation patterns, training, and testing with 10 fold cross-validation. The remaining 40% was held out from the initial training and test phase so that once development of the system was complete, the software could be tested on an independent data set and the reproducibility of the initial performance results verified. The pipeline for generating the test data is described in the Methods section.

Since the absence of evidence for alternative splicing does not prove the existence of a constitutive exon, a constitutive exon is defined for evaluation purposes to be an exon from a gene with a single known isoform, where each splice site is supported by at least 5 ESTs (or other cDNAs) aligned with 95% identity or higher. The hypothesis is that these genes have sufficient expression evidence to predict the presence or absence of alternative splicing.

#### Sequence conservation

The training set confirmed that splice sites and protein coding sequence were conserved between *D. melanogaster *and each of the three informant species. 99% of the constitutive di-nucleotide splice sites (AG and GT) annotated in *D. melanogaster *were found in the matching aligned informant sequence. Alternative splice sites were less frequently conserved, but only by a small degree, with over 95% of alternative splice sites found in the matched informant species. Table 1 shows the percentage of exons with matches to each of the informant species missing a splice site categorized by exon type. Exons with multiple duplicate functional splice sites (MS and IR exons) less frequently shared all splice sites with the informant species. In *D. simulans *for example, 12% of the multiple splice site exons (MS in Table 1) and 8% of the exons with retained introns (IR in Table 1) were missing a splice site. The lack of conservation in alternative splicing in nearly every case affects only one exon isoform leaving another shared exon isoform in place. In the vast majority of cases, the lack of observed conservation is not due to misalignments and missing sequence, although a small percentage of cases are affected by this problem.

**Table 1:** Percentage of *D. melanogaster *annotated exons missing at least one splice site in *D. simulans, D. yakuba *and *D. erecta*.

	*D. simulans*	*D. yakuba*	*D. erecta*
CS	2	1	1
CE	1	4	2
MS	10,1	9,0	11,0
IR	8,0	16,2	20,3

Percentages are organized by exon type: constitutive exons (CS), cassette exons (CE), exons with multiple splice sites (MS), and exons with intron retention (IR). The second number associated with the MS and IR rows is the percentage of exons where the non-conserved splice site is constitutive (used in all isoforms).

#### Prediction performance

ExAlt's prediction accuracy was measured on exons with an exon counted correct when the predicted left and right boundary matched the test exon. Internal exons begin with an acceptor and end with a donor. Initial exons begin with a transcription or translation start site and end with a donor site. Terminal exons begin with an acceptor and end with a transcription or translation stop site. Single exons begin with a transcription or translation start site and end with the transcription or translation stop site. (Single exons in the test were of the intron retention splicing type.) Sensitivity (the percentage of the test exons correctly detected) and specificity (the percentage of predicted exons, which match the test set) were used to measure performance.

Table 2 shows ExAlt's performance on the hold out set compared to the union of different publicly available gene predictions and an initial known exon given as input. This tests the ability to improve an existing annotation, where an initial exon and reading frame are known. Since many test sequences contained multiple overlapping exons, one exon was chosen at random and used as input. Experiments were repeated 10 times and the average taken. Results are listed in Table 2 as ExAlt-Exon for the ExAlt predictions informed by cross-species sequence conservation. Exon sensitivity and specificity are high since at least one predicted exon matched the test exon. For example, in the case of multiple splice site exons with two overlapping exons, a "naive" program predicting only the input exon would achieve 50% sensitivity and 100% specificity. When only a single exon isoform exists the naive program achieves 100% sensitivity and specificity respectively. For the results in Table 2 it was important to compare the decrease in specificity from the naive method in cases where only a single exon isoform occurs versus the gains in sensitivity when multiple overlapping exons occur. Two *ab initio *single isoform gene finders were included in the comparison, Augustus [34] and SNAP [35]. Also included is the single isoform gene finder, N-SCAN [36], which uses cross-species conservation with *Drosophila yakuba, Drosophila pseudoobscura*, and *Anopheles gambiae *[37].

**Table 2:** Prediction performance of ExAlt. Sensitivity (Sens) and Specificity (Spec) are shown for exons.

	Constitutive		Cassete		Multiple Splice		Intron Retention		All Exons	
	Sens	Spec	Sens	Spec	Sens	Spec	Sens	Spec	Sens	Spec
ExAlt-Exon	100	**96**	100	**89**	67	**94**	**61**	**89**	**84**	**94**
N-SCAN-Exon Union	100	86	100	**89**	65	79	55	78	82	84
Augustus-Exon Union	100	82	100	81	63	77	52	73	81	79
SNAP-Exon Union	100	77	100	79	64	74	51	76	81	77
SNAP+N-SCAN-Exon Union	100	73	100	74	69	68	57	70	83	72
Augustus+N-SCAN-Exon Union	100	79	100	77	68	73	57	68	83	76
Aug.+SNAP+N-SCAN-Exon Union	100	70	100	69	**71**	64	60	64	**84**	67

Columns are organized by exon type: Constitutive, Cassette, Multiple Splice, Intron Retention, and all exons counted together (All Exons). Row 1 shows ExAlt performance using an input exon and default parameter settings (ExAlt-Exon). Rows 2–6 show the union of different combinations of three gene finders (N-SCAN, SNAP, and Augustus) plus the input exon. (Aug. = Augustus)

The coordinates for start and stop codons were included as input to ExAlt but were excluded from input to the gene finders, making it potentially more difficult for the gene finders to accurately predict initial, terminal and single exons. Therefore, for the initial exons to be counted correct, a gene finder was only required to correctly predict the donor site. For terminal exons to be counted correct, a gene finder was only required to correctly predict the acceptor site, and for single gene exons to be counted correct, a gene finder only needed to predict an overlap with the known single exon. The gene finders were run on longer stretches of genomic sequence than ExAlt and have the added challenging task of determining gene boundaries. A gene finder may predict an initial, terminal or single exon to overlap an internal exon in the test set, which would be counted as an incorrect exon prediction. If the start and stop codon information were integrated into the gene finder prediction process, individual prediction performance for the respective gene finders would likely improve. However, since considerable effort has been taken to carefully train and tune the gene finders for annotating long stretches of genomic sequence, the current predictions serve as a reasonable baseline for measuring differences in prediction performance. Using the input exon plus the union of all three single isoform gene finders yields more of the correct multiple splice site exons (71% versus ExAlt's 67%) but at the cost of a large reduction in specificity (64% versus ExAlt's 94%). In the other cases, however, ExAlt matches or improves on the performance of the union of multiple gene finders.

Table 3 compares the prediction performance of ExAlt-Exon in Table 2 to ExAlt predictions using different parameter settings. The impact of using the gene structure information as input (ExAlt-Exon) was compared to alternatives shown in Table 3 as ExAlt-Frame and ExAlt-Default. ExAlt-Frame makes predictions without using exon coordinates as input but is limited to predicting exons that maintain reading frame consistency with the rest of the known gene. ExAlt-Default is given no gene structure information and checks all three possible reading frames before selecting the exons from the highest scoring reading frame. As expected, starting with an initial known exon improved overall performance, but even when gene structure information is precluded from input, a majority of the exon coordinates were correctly recovered (67% overall).

**Table 3:** Exon prediction accuracy using different ExAlt parameter settings.

	Constitutive		Cassete		Multiple Splice		Intron Retention		All Exons	
	Sens	Spec	Sens	Spec	Sens	Spec	Sens	Spec	Sens	Spec
ExAlt-Exon	100	96	100	89	67	94	61	89	84	94
Ex Alt-Exon- *ab initio*	100	88	100	85	69	83	70	87	87	84
ExAlt-Frame	96	95	70	80	53	87	48	82	72	89
Ex Alt- Frame- *ab initio*	97	87	72	74	56	76	48	80	74	82
ExAlt-Frame-Single	96	97	69	85	45	92	31	92	66	94
ExAlt-Default	89	84	58	63	49	77	43	74	67	79
Ex Alt-Default-*ab initio*	89	75	58	55	50	67	36	58	65	69
ExAlt-Default-Single	89	90	56	66	41	84	28	83	61	85
N-SCAN	87	84	51	80	33	66	31	66	57	78
Augustus	75	77	27	53	27	59	26	57	47	69
SNAP	76	72	42	61	29	56	27	62	50	67

Columns are organized by exon type: Constitutive, Cassette, Multiple Splice, Intron Retention, and all exons counted together (All Exons). Rows 1–2 show ExAlt performance using an input exon and default parameters from Table 2 (ExAlt-Exon) and no informant species (ExAlt-Exon-*ab initio*). Rows 3–5 show ExAlt performance using an input coding frame with default parameters (ExAlt-Frame), no informant species (ExAlt-Frame-*ab initio*), and at most 1 exon predicted per test sequence (ExAlt-Frame-Single). Rows 6–8 show ExAlt performance using no gene structure information with default parameters (ExAlt-Default), no informant species (ExAlt-Default-*ab initio*), and at most 1 exon prediction per test sequence (ExAlt-Default-Single). Output is shown for three single isoform gene finders N-SCAN, Augustus, and SNAP.

ExAlt-Exon, ExAlt-Frame, and ExAlt-Default were compared to the respective *ab initio *equivalent: ExAlt-Exon-*ab initio*, ExAlt-Frame-*ab initio*, and ExAlt-Default-*ab initio*. Each *ab initio *version is the GHMM equivalent to the PGHMM using only the target *D. melanogaster *sequence as input. The multi-species versions of ExAlt in all cases reduced the number of false positive predictions over the equivalent *ab initio *version, with little or no reduction in sensitivity.

Finally, the trade off between predicting multiple overlapping exons versus predicting at most one exon per test sequence was measured. With the hold out set comprised of 57% constitutive exons, 18% MS exons, 17% SE exons, and 9% IR exons, both single exon prediction versions of ExAlt (ExAlt-Frame-Single and ExAlt-Default-Single) captured a large percentage of the exons by simply correctly predicting one exon per sequence. When ExAlt is given the coding frame and restricted to predict at most one exon, an exon is correctly predicted in 94% of the sequences (ExAlt-Frame-Single in Table 3). Allowing ExAlt to predict overlapping exons (ExAlt-Frame in Table 3) lowered specificity to 89% but increased the number of correctly annotated exons to 72%. The last three rows show single isoform gene finding performance for N-SCAN, Augustus, and SNAP, which provided an additional point of reference to measure how well conventional gene finders performed in the evaluated gene regions.

**Table 4:** ExAlt results on the initial training and testing set in percentages.

	All Exons	
	Sens	Spec
ExAlt-Exon	82/-2	94/-1
ExAlt-Exon-*ab initio*	84/-3	86/+2
N-SCAN-Exon Union	82/0	82/-2
Augustus-Exon Union	81/0	81/+2
SNAP-Exon Union	81/0	77/0
SNAP+N-SCAN-Exon Union	83/0	72/0
Augustus+N-SCAN-Exon Union	83/0	75/-1
Aug.+SNAP+N-SCAN-Exon Union	84/0	68/+1
ExAlt-Frame	70/-2	87/-2
ExAlt-Frame-*ab initio*	72/-2	79/-3
ExAlt-Frame-Single	65/-1	91/-3
ExAlt-Default	65/0	78/-1
Ex Alt-Default *-ab initio*	65/0	66/-3
ExAlt-Default-Single	60/-1	84/-1
N-SCAN	56/-1	76/-2
Augustus	47/0	71/+2
SNAP	49/-1	67/0

Included next to each measurement is the difference in percentage points compared to performance in the held out set in Table 2 and Table 3. (Aug. = Augustus)

Prediction performance was much higher in constitutive exons than the other categories of alternatively spliced exons. Lack of sequence conservation partly explained the decrease in specificity. The highest specificity levels in the training set were found to occur when the two informant species *D. yakuba *and *D. erecta *were available. In the hold out set, 86% of the constitutive exons matched to *D. yakuba *and *D. erecta *sequences compared with 75% of the alternatively spliced exons. (In the remaining cases some other combination of one or two informant species were found.) Thus, in some cases ExAlt could not optimally use evidence of sequence conservation to limit false positive predictions. The *ab initio *versions of ExAlt (ExAlt- Frame-*ab initio *and ExAlt-Default-*ab initio*) also got a smaller percentage of cassette exons correct compared to constitutive exons. Many of the cassette exons were less than 100 bases long and the single species ExAlt (ExAlt-Frame-*ab initio *in Table 3) correctly identified a majority of these exons (62%). However, short exons (less than 100 bases) made up 69% of the cases where single species ExAlt did not get both splice sites exactly correctly. In contrast, all but 1 of the short constitutive exons were correctly identified.

To ensure that the results in the hold out set represent performance that is expected to be repeatable on similarly randomly distributed data sets, performance numbers for the original training set were examined using 10-fold cross validation and are shown in Table 4. Performance results among the two test sets differ in sensitivity and specificity by at most 3%.

## Conclusion

Single isoform gene structure input improved precision of ExAlt predictions. ExAlt reported overlapping false positive exons in only 4% of the constitutive exons (87% of the remaining exons were correctly classified as constitutive exons), while identifying nearly two thirds of the overlapping alternatively spliced exons. With limited gene structure information, ExAlt detected overlapping exons with modest increases in false positive predictions. Limiting over prediction in a non-expression based alternative exon finder proved to be a challenge since multiple overlapping sequence intervals are potentially predicted as exons. The problem is illustrated by the performance of the *ab initio *versions of ExAlt, which correctly predicted overlapping exons, but at the expense of increased over prediction of splice sites. Supplementing the statistical sequence models with evidence from cross-species sequence conservation proved to be an effective strategy in reducing the number of false positive predictions, while maintaining sensitivity levels. 

In model species such as *Drosophila melanogaster*, significant effort has gone into providing accurate gene structure annotations, which account for many of the proteins present in the cell. With the abundance of alternative splicing already known to occur, it is likely that evidence for new examples of alternative splicing will continue to grow. Therefore, even with the availability of high quality annotations, there are new variations in gene structure yet to be discovered and non-expression based prediction methods such as ExAlt can be used to search for new cases of alternative splicing.

## Methods

### Data preparation

Test exons were downloaded from FlyBase [38] using *Drosophila melanogaster *annotation version 4.2.1. Each gene locus was partitioned into non-overlapping intervals, with each interval containing all annotated overlapping exons. Test exons were selected from transcripts annotated with the same start/stop codon pair with splice sites located in the coding region. In a few cases, multiple overlapping test exons are spliced to neighboring exons in such a way that portions of the known exon sequence contain multiple functional overlapping reading frames. Currently ExAlt assumes the occurrence of a single reading frame, thus limiting its sensitivity in some cases. We plan to incorporate explicit prediction of multiple overlapping reading frames in the near future, to further improve sensitivity on the test set.

An initial set of 606 regions annotated with alternative splicing were searched for duplicate sequences. WU-blastn 2.0 [39] was used for an 'all against all' search to remove repeat sequence when two exons match with an E-value < 10^-21^, leaving 600 exon regions. Each remaining non-redundant exon region was extracted from the originating genome location with flanking intron sequence of 400 bases (or the length of the adjacent intron, whichever is shorter). 400 was chosen as a cutoff to limit the potential for aligning long stretches of poorly conserved intron sequence, while maintaining reasonably long stretches of sequence to predict alternative splicing patterns. WU-blastn 2.0 is used to find potential homologs in the three informant species *D. simulans*, *D. yakuba*, and *D. erecta*. The *D. simulans *genome was downloaded from the UCSC genome browser [40]. The *D. yakuba *and *D. erecta *genomes were downloaded from [41]. (*D. simulans *and *D. yakuba *sequence was generated by the Genome Sequencing Center, WUSTL School of Medicine and *D. erecta *sequence was generated by Agencourt.) The best matching sequence with an E-value < 10^-19 ^was retained along with 50 bases of flanking sequence for input to the multiple sequence alignment program muscle [42]. All aligned sequences were required to differ in length by at most 10% to the query *D. melanogaster *sequence. N-SCAN and Augustus predictions were downloaded from the UCSC Genome browser [43,44] and SNAP predictions were downloaded from [45]. Branch lengths were obtained from [46].

### Prediction model

**Figure 5:**
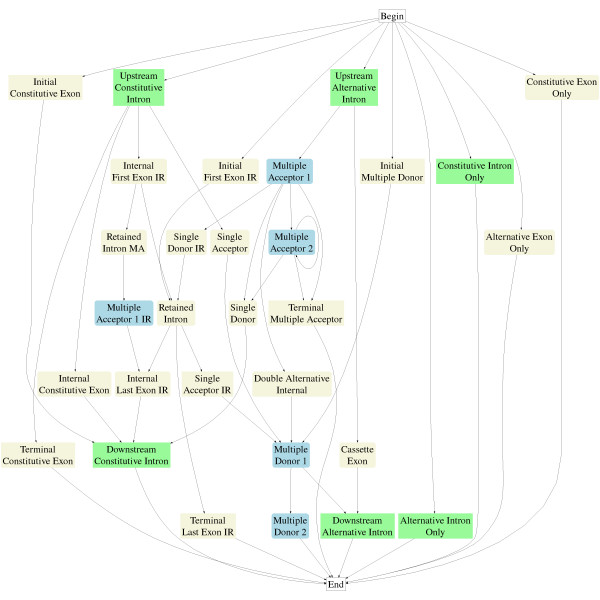
Model from Figure 3 expanded to include alternative splicing in terminal and initial exons and candidate single exon genes. Blue and beige states reflect possible protein coding exons (beige) or partial protein coding exons (blue) and represent three states for each state shown, one for each of the three coding phases. Special states "Beg" and "End" show the respective begin and end states in the model.

Figure 5 shows the model used in performance evaluation. States are included to model splicing patterns in exons at the beginning and end of genes and to account for the three possible reading frames and to allow for the entire sequence to be a coding exon or intron. For example, "Alternative Exon Only" is the signal pair (Beg,END-EXON_*A*_) and emits a contiguous coding sequence with no splice sites. Some candidate alternative splicing events are excluded from Figure 5. For example, the interval between the two splice site signals *MDN-IR *and *MA1-IR *represents a retained intron between an upstream exon with multiple functional donor sites and a downstream exon with multiple functional acceptor sites. While such an alternative splicing pattern might occur (at least in theory), no examples were found in the training of ExAlt, thus, there is 0 probability of entering the state. There were 15 cases of "rare" alternative splicing types: 8 exons contained more than 2 donor or acceptor sites, 6 exons classified as intron retention cases contained multiple splice sites in one of the exon forms and 1 exon contained both multiple acceptor sites and multiple donor sites. Each exon form was included in the performance analysis. [47] gives a more complete survey of complex alternative splicing patterns.

Gaps in the multiple sequence alignment are treated as missing data. Maximum likelihood parameter estimation is used to set parameters for the 10 sequence models and state transitions. Training consists of iterating through labeled examples of alternatively spliced exons and constitutive exons. Substitution rates are defined by randomly selecting values within a range of 0.5 to 2.5 and adjusting the value to maximize the F-score (=(2 × *Sn *× *Sp*)/(*Sn *+ *Sp*)) in the training set, where sensitivity (Sn) is the percentage of protein coding nucleotides correctly labeled and specificity (Sp) is the percentage of predicted protein coding nucleotides correctly labeled.

## Availability

ExAlt is implemented in C++ and freely available for download as an open source package from the ExAlt web page [48]. All data used in this study is available for download from the ExAlt web page.

## Competing interests

The author(s) declare that they have no competing interests.

## Authors' contributions

JEA designed and implemented ExAlt and wrote the manuscript. SLS contributed to test and feature design and helped with writing the manuscript.

## References

[B1] Johnson JM, Castle J, Garrett-Engele P, Kan Z, Loerch PM, Armour CD, Santos R, Schadt EE, Stoughton R, Shoemaker DD (2003). Genome-wide survey of human alternative pre-mRNA splicing with exon junction microarrays. Science.

[B2] Maniatis T, Tasic B (2002). Alternative pre-mRNA splicing and proteome expansion in metazoans. Nature.

[B3] Cartegni L, Chew SL, Krainer AR (2002). Listening to silence and understanding nonsense: exonic mutations that affect splicing. Nature Reviews Genetics.

[B4] Mironov AA, Fickett JW, Gelfand MS (1999). Frequent alternative splicing of human genes. Genome Research.

[B5] Brett D, Hanke J, Lehmann G, Haase S, Delbruck S, Krueger S, Reich J, Bork P (2000). EST comparison indicates 38% of the human mRNAs contain possible alternative splice forms. FEBS Letters.

[B6] Croft L, Schandorff S, Clark F, Burrage K, Arctander P, Mattick JS (2000). ISIS, the intron information system, reveals the high frequency of alternative splicing in the human genome. Nature genetics.

[B7] Kan Z, Rouchka EC, Gish WR, States DJ (2001). Gene structure prediction and alternative splicing analysis using genomically aligned ESTs. Genome Research.

[B8] Modrek B, Resch A, Grasso C, Lee C (2001). Genome-wide detection of alternative splicing in expressed sequences of human genes. Nucleic Acids Research.

[B9] Haas BJ, Delcher AL, Mount SM, Wortman JR, Smith RK, Hannick LI, Maiti R, Ronning CM, Rusch DB, Town CD, Salzberg SL, White O (2003). Improving the Arabidopsis genome annotation using maximal transcript alignment assemblies. Nucleic Acids Res.

[B10] Carninci P, Kasukawa T, Katayama S, Gough J, Frith MC, Maeda N, Oyama R, Ravasi T, Lenhard B, Wells C, Kodzius R, Shimokawa K, Bajic VB, Brenner SE, Batalov S, Forrest ARR, Zavolan M, Davis MJ, Wilming LG, Aidinis V, Allen JE, Ambesi-Impiombato A, Apweiler R, Aturaliya RN, Bailey TL, Bansal M, Baxter L, Beisel KW, Bersano T, Bono H, Chalk AM, Chiu KP, Choudhary V, Christoffels A, Clutterbuck DR, Crowe ML, Dalla E, Dalrymple BP, de Bono B, Gatta GD, di Bernardo D, Down T, Engstrom P, Fagiolini M, Faulkner G, Fletcher CF, Fukushima T, Furuno M, Futaki S, Gariboldi M, Georgii-Hemming P, Gingeras TR, Gojobori T, Green RE, Gustincich S, Harbers M, Hayashi Y, Hensch TK, Hirokawa N, Hill D, Huminiecki L, Iacono M, Ikeo K, Iwama A, Ishikawa T, Jakt M, Kanapin A, Katoh M, Kawasawa Y, Kelso J, Kitamura H, Kitano H, Kollias G, Krishnan SPT, Kruger A, Kummerfeld SK, Kurochkin IV, Lareau LF, Lazarevic D, Lipovich L, Liu J, Liuni S, McWilliam S, Babu MM, Madera M, Marchionni L, Matsuda H, Matsuzawa S, Miki H, Mignone F, Miyake S, Morris K, Mottagui-Tabar S, Mulder N, Nakano N, Nakauchi H, Ng P, Nilsson R, Nishiguchi S, Nishikawa S, Nori F, Ohara O, Okazaki Y, Orlando V, Pang KC, Pavan WJ, Pavesi G, Pesole G, Petrovsky N, Piazza S, Reed J, Reid JF, Ring BZ, Ringwald M, Rost B, Ruan Y, Salzberg SL, Sandelin A, Schneider C, Schonbach C, Sekiguchi K, Semple CAM, Seno S, Sessa L, Sheng Y, Shibata Y, Shimada H, Shimada K, Silva D, Sinclair B, Sperling S, Stupka E, Sugiura K, Sultana R, Takenaka Y, Taki K, Tammoja K, Tan SL, Tang S, Taylor MS, Tegner J, Teichmann SA, Ueda HE, van Nimwegen E, Verardo R, Wei CL, Yagi K, Yamanishi H, Zabarovsky E, Zhu S, Zimmer A, Hide W, Bult C, Grimmond SM, Teasdale RD, Liu ET, Brusic V, Quackenbush J, Wahlestedt C, Mattick JS, Hume DA, Kai c, Sasaki D, Tomaru Y, Fukuda S, Kanamori-Katayama M, Suzuki M, Aoki J, Arakawa T, lida J, Imamura K, Itoh M, Kato T, Kawaji H, Kawagashira N, Kawashima T, Kojima M, Kondo S, Konno H, Nakano K, Ninomiya N, Nishio T, Okada M, Plessy C, Shibata K, Shiraki T, Suzuki S, Tagami M, Waki K, Watahiki A, Okamura-Oho Y, Suzuki H, Kawai J, Hayashizaki Y, The FANTOM Consortium, RIKEN Genome Exploration Research Group and Genome Science Group (Genome Network Project Core Group) (2005). The transcriptional landscape of the mammalian genome. Science.

[B11] Modrek B, Lee C (2002). A genomic view of alternative splicing. Nature Genetics.

[B12] Xu Q, Lee C (2003). Discovery of novel splice forms and functional analysis of cancer-specific alternative splicing in human expressed sequences. Nucleic Acids Res.

[B13] Sorek R, Ast G (2003). Intronic sequences flanking alternatively spliced exons are conserved between Human and Mouse. Genome Research.

[B14] Sorek R, Shemesh R, Cohen Y, Basechess O, Ast G, Shamir R (2004). A non-EST based method for exon-skipping prediction. Genome Research.

[B15] Dror G, Sorek R, Shamir R (2005). Accurate identification of alternatively spliced exons using support vector machine. Bioinformatics.

[B16] Yeo GW, Nostrand EV, Holste D, Poggio T, Burge CB (2005). Identification and analysis of alternative splicing events conserved in human and mouse. PNAS.

[B17] Rätsch G, Sonnenburg S, Scholköpf B (2005). RASE: recognition of alternatively spliced exons in C. elegans. Bioinformatics.

[B18] Philipps DL, Park JW, Graveley BR (2004). A computational and experimental approach toward a priori identification of alternatively spliced exons.

[B19] Cawley SL, Pachter L (2003). HMM sampling and applications to gene finding and alternative splicing. Bioinformatics.

[B20] Alexandersson M, Cawley S, Pachter L (2003). SLAM: cross-species gene finding and alignment with a generalized pair hidden Markov model. Genome Research.

[B21] Hiller M, Backofen R, Heymann S, Busch A, Glaber TM, Freytag JC (2004). Efficient prediction of alternative splice forms using protein domain homology. In Silico Biol.

[B22] Hiller M, Huse K, Platzer M, Backofen R (2005). Non-EST based prediction of exon skipping and intron retention events using Pfam information. Nucleic Acids Res.

[B23] Ohler U, Shomron N, Burge CB (2005). Recognition of unknown conserved alternatively spliced exons. PLOS Comp Bio.

[B24] Allen JE, Salzberg SL (2005). JIGSAW: integration of multiple sources of evidence for gene prediction. Bioinformatics.

[B25] Pedersen JS, Hein J (2003). Gene finding with a hidden Markov model of genome structure and evolution. Bioinformatics.

[B26] Siepel A, Haussler D (2003). Combining phylogenetic and hidden Markov models in biosequence analysis. Proceedings of the Seventh Annual International Conference on Computational Molecular Biology (RECOMB 2003).

[B27] McAuliffe JD, Pachter L, Jordan MI (2004). Multiple-sequence functional annotation and the generalized hidden Markov phylogeny. Bioinformatics.

[B28] Boffelli D, McAuliffe J, Ovcharenko D, Lewis KD, Ovcharenko I, Pachter L, Rubin EM (2003). Phylogenetic shadowing of primate sequences to find functional regions of the human genome. Science.

[B29] Sugnet CW, Kent WJ, Ares M, Haussler D (2004). Transcriptome and genome conservation of alternative splicing events in humans and mice. Pacific Symposium on Biocomputing.

[B30] Black DL (2003). Mechanisms of alternative pre-messenger RNA splicing. Annu Rev Biochem.

[B31] Yeo GWM (2005). Splicing regulators: targets and drugs. Genome Biology.

[B32] Hasegawa M, Kishino H, Yano T (1985). Dating the human-ape splitting by a molecular clock of mitochondrial DNA. J Mol Evol.

[B33] Felsenstein J (1981). Evolutionary trees from DNA sequences: a maximum likelihood approach. J Mol Evol.

[B34] Stanke M, Waack S (2003). Gene prediction with a hidden Markov model and a new intron submodel. Bioinformatics.

[B35] Korf I (2004). Gene finding in novel genomes. BMC Bioinformatics.

[B36] Gross SS, Brent MR (2005). Using multiple alignments to improve gene prediction. RECOMB.

[B37] N-SCAN UCSC Genome Browser page. http://genome.ucsc.edu/cgi-bin/hgTrackUi?hgsid=73664894%&c=chr2L&g=nscanGene.

[B38] Drysdale RA, Crosby MA, The FlyBase Consortium (2005). FlyBase: genes and gene models. Nucleic Acids Res.

[B39] WU-BLAST. http://blast.wustl.edu.

[B40] UCSC Genome Browser. ftp://hgdownload.cse.ucsc.edu/goldenPath/droSim1/chromosomes.

[B41] AAA Drosophila resource. http://rana.lbl.gov/drosophila/multipleflies.html.

[B42] Edgar RC (2004). MUSCLE: multiple sequence alignment with high accuracy and high throughput. Nucleic Acids Res.

[B43] N-SCAN predictions. http://hgdownload.cse.ucsc.edu/goldenPath/dm2/database/nscanGene.txt.gz.

[B44] Augustus predictions. http://hgdownload.cse.ucsc.edu/goldenPath/dm2/database/augustus.txt.gz.

[B45] Gilbert DG DroSpeGe, a public database of Drosophila species genomes. http://insects.eugenes.org/DroSpeGe/.

[B46] Pollard DA Drosophila trees. http://rana.lbl.gov/~dan/trees.html.

[B47] Nagasqaki H, Arita M, Nishizawa T, Suwa M, Gotoh O (2005). Species-specific variation of alternative splicing and transcriptional initiation in six eukaryotes. Gene.

[B48] ExAlt web page. http://www.cbcb.umd.edu/software/exalt.

